# Evaluation and comparison of recombinase polymerase amplification coupled with lateral-flow bioassay for *Escherichia coli* O157:H7 detection using diifeerent genes

**DOI:** 10.1038/s41598-021-81312-6

**Published:** 2021-01-21

**Authors:** Alka Rani, Vivek B. Ravindran, Aravind Surapaneni, Esmaeil Shahsavari, Nagalakshmi Haleyur, Nitin Mantri, Andrew S. Ball

**Affiliations:** 1 School of Science, RMIT University, Bundoora West, VIC 3083 Australia; 2 The Pangenomics Group, School of Science, RMIT University, Melbourne, VIC 3083 Australia; 3 South East Water, Frankston, VIC Australia; 4 ARC Training Centre for the Transformation of Australia’s Biosolids Resource, RMIT University, Bundoora West, VIC 3083 Australia

**Keywords:** Biological techniques, Biotechnology, Microbiology, Molecular biology, Diseases

## Abstract

Shiga toxin-producing *Escherichia coli* serotype O157:H7 is a food and waterborne zoonotic pathogen causing gastroenteritis in humans. Rapid and simple detection in water and food is imperative to control its spread. However, traditional microbial detection approaches are time-consuming, expensive and complex to operate at the point-of-care without professional training. We present a rapid, simple, sensitive, specific and portable method for detection of *E. coli* O157:H7 in drinking water, apple juice and milk. We evaluated the effect of gene selection in detecting *E. coli* O157:H7 using recombinase polymerase amplification coupled with a lateral flow assay using *rfbE, fliC* and *stx* gene targets. As low as 100 ag and 1 fg DNA, 4–5 CFU/mL and 10^1^ CFU/mL of *E. coli* O157:H7 was detected using the *stx* and *rfbE* gene targets respectively with 100% specificity, whilst the detection limit was 10 fg DNA and 10^2^ CFU/mL for the *fliC* gene target, with 72.8% specificity. The RPA-LFA can be completed within 8 min at temperatures between 37 and 42 °C with reduced handling and simple equipment requirements. The test threshold amplification of the target was achieved in 5–30 min of incubation. In conclusion, RPA-LFA represents a potential rapid and effective alternative to conventional methods for the monitoring of *E. coli* O157:H7 in food and water.

## Introduction

Shiga-toxin producing *Escherichia coli* (STEC) strain O157:H7, classified as an enterohemorrhagic *E. coli* (EHEC) is the most common pathogenic *E. coli*, which colonizes the human gastrointestine, damaging the intestinal wall lining causing bloody and non-bloody diarrhea and more severe diseases such as hemolytic uremic syndrome (HUS) and thrombotic thrombocytopenic purpura (TTP)^1,2^. According to the World Health Organization (WHO) (https://www.who.int/news-room/fact-sheets/detail/diarrhoeal-disease), in 2005, 1.8 million people died from diarrheal diseases, traced to the consumption of food contaminated with pathogens including *E. coli* O157:H7^3^. A systematic review conducted by Majowicz et al. (2014) on the global prevalence of STEC infections estimated that over 2.8 million people are infected every year. Globally, numeous outbreaks (approximately 350) related to *E. coli* O157:H7 occurred in 49 USA states, Canada, Great Britain, Japan and Ireland between 1982 and 2006^4^. More than half of these outbreaks (54%) originated from contaminated water and food^1^, confirming that *E. coli* O157:H7 poses a risk to public health and wellbeing.

Prompt diagnosis of *E. coli* O157:H7 is the most effective strategy for monitoring and managing the disease (https://www.cdc.gov/ecoli/). However traditional diagnoses relies on culturing the STEC on a selective media such as sorbitol MacConkey agar (SMAC) or Laural Tryptose broth -4-methylumbelliferyl-b-d-glucuronide (LTB-MUG)^5^. These methods are largely accurate; however they are time-consuming (5–7 days), expensive and labour-intensive. Consequently, these methods are unsuitable for real-time monitoring of any pathogen outbreak. Molecular assays such as polymerase chain reaction (PCR)^6^ and pulse field gel electrophoresis (PFGE)^7^ have been developed for the identification of *E. coli* O157:H7. These methods overcome the limitation of culture based methods, having good sensitivity and specificity; however these techniques require sophisticated devices, trained personnel and are generally expensive. Furthermore they are unsuitable for point-of-care (POC) use. Enzyme linked immunosorbent assay (ELISA), which relies on the detection of antigens with specific antibodies has POC application; however, low sensitivity and cross-reactivity are associated with this approach devaluing its application^8,9^.

Lateral flow (LF) paper-based microfluidics (LFPBM) is an emerging detection device that fulfils the criteria of an ideal POC diagnostic tool given by the World Health Organization (WHO) as ASSURED: Affordable, Sensitive, Specific, User-friendly, Rapid and Robust, Equipment-free and Deliverable^10^. The principle of these LF devices is the capillary action of fluids through 3-dimensional porous microstructures. The LF method consists of an easy design and results in rapid visual outcomes when compared to other detection approaches like microfluidic analytical devices^11,12^. A paper-based sandwich ELISA method has been developed for *E. coli* O157:H7 using primary antibodies, horseradish peroxidase (HRP) conjugated anti-goat secondary antibodies and tetra-methylbenzidine-hydrogen peroxide (TMB-H_2_O_2_). Although the specificity was found satisfactory and the detection limit was 1 × 10^4^ CFU/mL, the complete assay takes 3–4 h and requires multiple antibodies, making the assay 3–4 times more expensive than traditional culture based and molecular methods^13^.

Molecular techniques are however rapid, sensitive, specific, efficient and robust^14^. In addition, recently, isothermal amplification approaches have gained attention over PCR-based molecular approaches for on-site diagnosis. Loop-mediated isothermal amplification (LAMP) has been developed for *E. coli* O157:H7 but multiple incubations at high temperatures (60 °C and 80 °C) for 1 h and the requirement for the use of six primers represent significant limitations for this method^15^. Recombinase polymerase amplification (RPA) overcomes the limitations of LAMP, requiring incubation at a constant, mesophilic temperature (37–42 °C) for less than half an hour. Recombinase polymerase amplification eradicates the requirement for heat cycles, yet can carry out exponential amplification to generate millions of copies of the target gene with one primer set^16^. Therefore, a combination of RPA and paper-based lateral flow assay (LFA) offers significant promise for use in POC detection of pathogens; we recently developed a POC method to detect helminth ova in wastewater using RPA-LFA^17^. In terms of *E. coli* O157:H7, Hu et al. recently reported the application of RPA combined with a dipstick for the detection of the pathogen in raw milk samples. The specificity and sensitivity reported was extremely good, with detection of 1 fg of *E. coli* O157:H7 genomic DNA^18^. Although the selection of the gene target for any RPA assay is crucial^19^, to date there are no publications which has evaluated the effect of gene target selection in detecting *E. coli* O157:H7 using RPA-LFA. To fill this knowledge gap, we aimed to extend work in the field of RPA application by evaluating the effect of gene target selection on RPA amplification coupled with LFA. We assessed the application of *rfbE*, *fliC* and *stx* genes of *E. coli* O157 :H7 as targets for detection through RPA-LFA. Moreover, validation of RPA-LFA on more commercial samples such as drinking water and apple juice brings further novelty to this study. Test threshold amplification by RPA was achieved in 5 min at 37–42 °C with no false negative results. RPA-LFA has variable sensitivity and speficity with varying gene targets. This LF-based detection strategy eliminates the need for purification and visualisation of the amplicon through gel electrophoresis, significantly reducing detection time. RPA-LFA can be used as a high-throughput approach alternative to routinely used conventional laboratory methods and also has the potential to be transferred into field-deployable tests for surveillance of *E. coli* O157:H7 outbreaks.

## Results

### Designing and screening of primers

**Figure 1:**
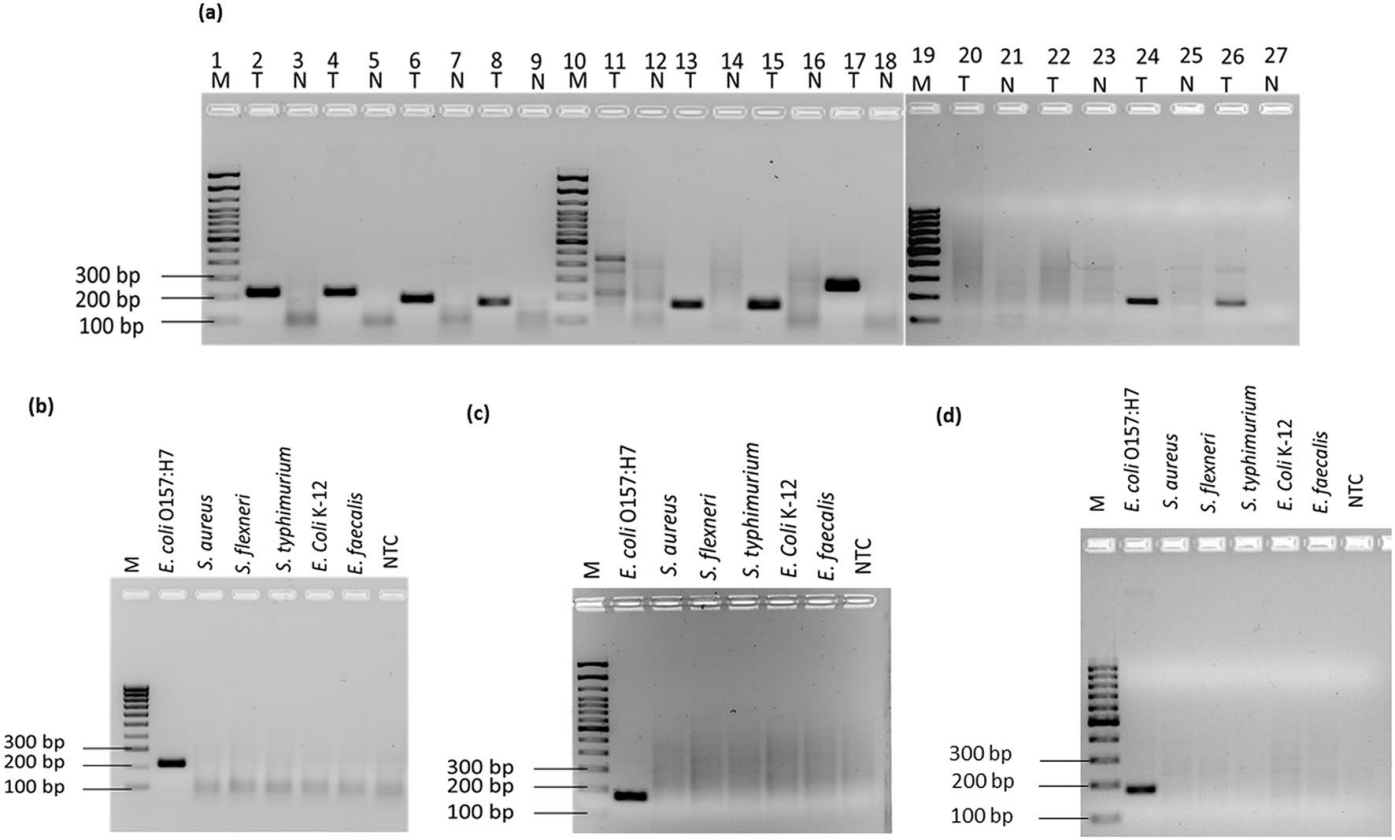
Primer screening and specificity test (**a**) Primer selection: Lane 1, 10 and 19: 100 bp molecular marker, Lane 2 and 3: rfbE F1/R1; lane 4 and 5: rfbE F2/R2; lane 6 and 7: rfbE F3/R3; lane 8 and 9: rfbE F4/R4; lane 11 and 12: fliC F1/R1; lane 13 and 14: fliC F2/R2; lane 15 and 16: fliC F3/R3; lane 17 and 18: fliC F4/R4; lane 20 and 21: stx1 F1/R1; lane 22 and 23: stx1 F2/R2; lane 24 and 25: stx2 F1/R1; lane 26 and 27: stx2 F2/R2. Specificity Assay **(b)** Amplification based on the rfbE F1/R1 primer set, (**c**) Amplification based on the fliC F2/R2 primer set and (**d**) Amplification based on the stx2 F1/R1 primer set—with RPA-AGE using *E. coli* O157:H7, *Staphylococcus aureus*,* Shigella flexneri*,* Salmonella thyphimurium*, *E. coli* K-12, *Enterococcus faecalis*, non-template control (NTC). *M* molecular marker, *T* template, *N* non-template control.

PrimedRPA software was selected for primer design because of its specification for RPA primers, with all filters set by default. Following design, primers were assessed through National Center for Biotechnology Information—Basic Local Alignment Sequence Tool (NCBI-BLAST) and OligoAnalyzer Tool (IDT) and screened depending on their binding score to non-target domains^20^. A list of all the screened primers for *rfb*E, *stx* and *fli*C gene targets is shown in Table 1. Out of a total 12 screened primer sets, 9 were able to amplify the amplicon as predicted from in silico analysis (Fig. 1a). The RPA-AGE results confirmed the amplification of target *E. coli* O157:H7 with rfbE F1/R1, rfbE F2/R2, rfbE F3/R3, rfbE F4/R4, fliC F2/R2, fliC F3/R3, fliC F4/R4, Eco stx2 F1/R1, Eco stx F2/R2 primer sets; no amplification was attained with fliC F1/R, stx1 F1/R1 and stx1 F2/R21. Out of these rfbE F1/R1, fliC F2/R2 and stx2 F1/R1 were found optimal primer sets due to an absence of non-specific bands in the template as well as non-template control (NTC) (Fig. 1a).

**Table 1:** A list of all primers tested in this study.

Primer/probe	Sequence (5′–3′)	Primer/probe length	Amplicon size
**For fliC gene**			
Eco fliC F1	TTGATGAAATTGACCGCGTATCCGGCCAGACC	32 nt	172
Eco fliC R1	ATTTACGTTAAAGCCATTCAGACCCAGAGTAT	32 nt	
Eco fliC F2	TGCAGGCAACTTGACGACTAACAACGCTGGTAG	33 nt	169
Eco fliC R2	TTAACGGAGCTACTGGAGTGGTTGTCGCAGG	31 nt	
Eco fliC F3	ATGAAATTGACCGCGTATCCGGCCAGACCCAG	32 nt	169
Eco fliC R3	ACCATTTACGTTAAAGCCATTCAGACCCAGAG	32 nt	
Eco fliC F4	CTTTATGATCTGAAAACCGAAAATACCTTGTT	32 nt	266
Eco fliC R4	TCTTTTGTGGTGTAAGAACCATTAACAGATTT	32 nt	
**For rfbE gene**			
Eco rfbE F1	AGCTTTGTTAGCGTTAGGTATATCGGAAGGAGA	33 nt	216
Eco rfbE R1	ACATGGATGTCCGTATAAATGGACACACATAAT	33 nt	
Eco rfbE F2	AGCTTTGTTAGCGTTAGGTATATCGGAAGGAGATG	35 nt	217
Eco rfbE R2	TCACATGGATGTCCGTATAAATGGACACACATAAT	35 nt	
Eco rfbE F3	GTTAACTGTTATGTTGTACTGCTTCATTTTTA	32 nt	187
Eco rfbE R3	GAGTCCAGACATTCATTTACATATTCTTTTTC	32 nt	
Eco rfbE F4	AAAGAGAGGAATTAAGGAATCACCTTGCAGAT	32 nt	169
Eco rfbE R4	ATTCGATAGGCTGGGGAAACTAGGTAAATTAA	32 nt	
**For stx gene**			
Eco stx1 F1	GGAACCTCACTGACGCAGTCTGTGGCAAGAGC	32	179
Eco stx1 R1	CAACCTTCCCCAGTTCAATGTAAGATCAACAT	32	
Eco stx1 F2	CCTCACTGACGCAGTCTGTGGCAAGAGCGATG	33	179
Eco stx1 R2	TACTCAACCTTCCCCAGTTCAATGTAAGATCA	32	
Eco stx2 F1	GTGGCCGGGTTCGTTAATACGGCAACAAATAC	32	179
Eco stx2 R1	TGAAACCAGTGAGTGACGACTGATTTGCATTC	32	
Eco stx2 F2	ATATCTCAGGGGACCACATCGGTGTCTGTTAT	32	180
Eco stx2 R2	GTAGAAAGTATTTGTTGCCGTATTAACGAACC	32	

*FP* forward primer, *RP* reverse primer, *nt* nucleotides.

### Evaluation of optimal conditions and validation of the primers

The selected primer sets (rfbE F1/R1, fliC F2/R2 and stx2 F1/R1) were optimized in terms of incubation temperature and time using the RPA-AGE assay. For rfbE F1/R1 and stx2F1/R1, DNA amplification occurred from 37 to 42 °C (Fig S1, S3) with test threshold amplification beginning after 5 min and continuing for 30 min for rfbE F1/R1; in contrast, for stx2 F1/R1 threshold amplification was achieved only after 10 min (Fig. S4, S6). For the fliC F2/R2, amplification occurred between 38 and 42 °C; only a faint band was observed at 37 °C (Fig S2). Test threshold amplification resulted between 15 and 30 min incubation; limited amplification was observed in the first 10 min (Fig S4). Optimum incubation conditions for this study were chosen as 39 °C for 20 min regardless of gene type.

*E. coli* K-12, *Staphylococcus aureus*, *Salmonella typhimurium, Enterococcus faecalis* and *Shigella flexneri* were initially tested for cross-reactivity against the screened primers targeting the *rfb*E, *fli*Cand *stx* genes. For NTC control, NFW was used as the template. No cross-reaction was observed for non-specific target bacteria using rfbE F1/R1 (Fig. 1b), fliC F2/R2 (Fig. 1c) and stx2 F1/R1(Fig. 1d); all three primer sets were selective for *E. coli* O157:H7 identification.

### Development of LFA and its evaluation for selectivity

Following primer validation using the RPA-AGE assay, the selected primer sets were modified to develop the LF-bioassay (Table 2). Different labels were added to both primer sets and LF-probes. Primary experimental work was carried out using 10 ng DNA of *E. coli* O157:H7 and NTC. Three different RPA-LFA were established for all target genes, *rfb*, *fliC* and *stx*. Following amplification at 39^O^C for 20 min, 6 µL of amplified product mixed with 84 µL of extraction buffer (provided within the kit) from the PCRD nucleic acid detection kit and was loaded into the sample well. The control line (C) was coloured for all test samples and NTC; no false negative [coloured test line (2)] was observed in the presence of *E. coli* O157:H7 for all selected primer sets (rfbE F1/R1, fliC F2/R2 and stx2 F1/R1). The results show that the RPA-LFA targeting all genes were efficient in detecting *E. coli* O157:H7.

**Table 2:** A list of all primers and probes used in this study.

Primer/probe	Sequence (5′–3′)	Primer/probe length	Amplicon size
**Selected primers and probes for LFA**			
Eco fliC F2	TGCAGGCAACTTGACGACTAACAACGCTGGTAG	33 nt	169
Eco fliC R2	**Bt**-TTAACGGAGCTACTGGAGTGGTTGTCGCAGG	31 nt	
Eco fliC P2 probe	**5′-FAM**-TGCTCAAAGCAGCGAGCGAAGGTAGTGACGGTG-(**THFresidue)-**CTCTCTGACATTCAATGG-**C3 spacer-3′**	51 nt	
Eco rfbE F1	AGCTTTGTTAGCGTTAGGTATATCGGAAGGAGA	33 nt	216
Eco rfbE R1	**Bt**-ACATGGATGTCCGTATAAATGGACACACATAAT	33 nt	
Eco rfbE P1 probe	**5′-FAM-**GTTGATTCAGATAATGAAACTTGGCAAATGT-(**THFresidue**) TGTTAGTGACATAGAAC-**C3spacer-3′**	48 nt	
Eco stx2 F1	5′-GTGGCCGGGTTCGTTAATACGGCAACAAATAC-3′	32 nt	179
Eco stx2 R1	5′-Biotin-TGAAACCAGTGAGTGACGACTGATTTGCATTC-3′	32 nt	
Eco stx2 P1 probe	**5′-FAM-**AGTGCCCGGTGTGACAACGGTTTCCATGACAA**(THF residue)**GGACAGCAGTTATACCA -**C3spacer-3′**	49	

*Bt* biotin, *FAM* fluorescein, *THF* tetrahydrofuran residue, *nt* nucleotides.

**Figure 2:**
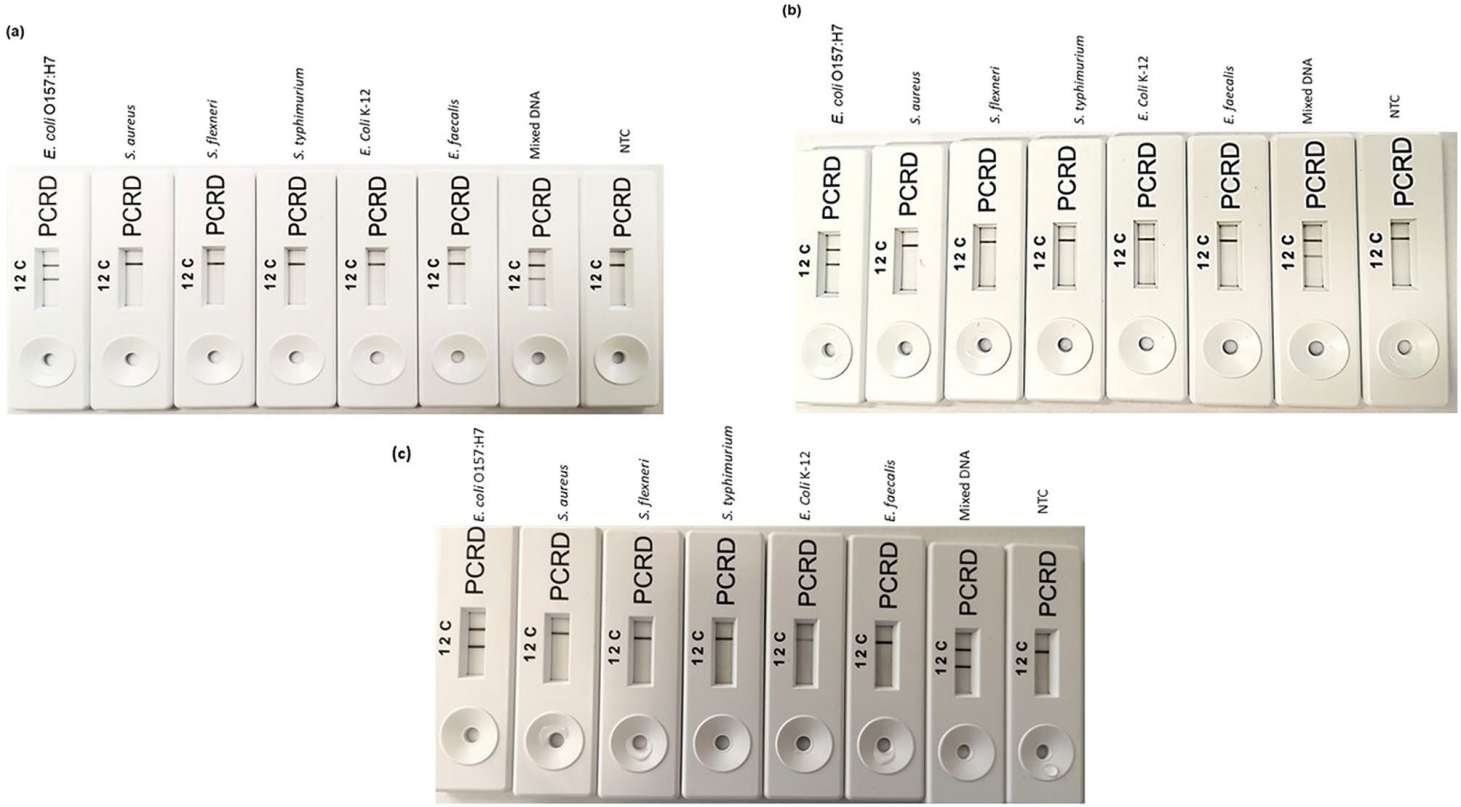
Validation of RPA-LFA for specificity. (**a**) Using the rfbE F1/R1 primer set (**b**) Using the fliC F2/R2 primer set, and, (**c**) Using the stx1 F2/R2 primer set. From left to right: *Escherichia coli* O157:H7, *Staphylococcus aureus*, *Shigella flexneri*, *Salmonella thyphimurium*, *E. coli* K-12, *Enterococcus faecalis*, mixed bacterial DNA, non-template control (NTC).

Other non-target bacterial strains such as *S. aureus*, *S. flexneri*, *S. typhimurium*, *E. coli* K-12, *E. faecalis* and a mixture of DNA from all bacteria were used at the same concentration as *E. coli* O157:H7 to validate the selectivity of the RPA-LFA. Nuclease free water (NFW) was also introduced in the selectivity test as the NTC. For RPA-LFA (targeting *rfbE*, *fliC* and *stx*), no visible coloured test line (2) was produced for all non STEC bacteria and NTC, whereas coloured test lines (2) was seen for *E. coli* O157:H7 and a mixture of *E. coli* O157:H7’s DNA (Fig. 2a–c). However, when further bacterial strains (Table 3) were used to assess speficity, the LFA targeting the *rfbE* and *stx* genes were found 100% specific in detecting the *E. coli* O157:H7. In contrast the LFA targeting the *fliC* gene was found only 72.8% selective for *E. coli* O157:H7 after producing coloured test line for 6 non-target bacterial strains (Table 3).

**Table 3:** Selectivity evaluation of RPA-AGE and RPA-LFA using *rfbE* and *fliC* gene targets.

S. no	Bacteria	Serotype	Strain	Classification	*rfbE* gene		*fliC* gene		*stx* gene	
					RPA-AGE	RPA-LFA	RPA-AGE	RPA-LFA	RPA-AGE	RPA-LFA
1	*E. coli*	O157:H7	ATCC 43888	STEC	+	+	+	+	+	+
2	*E. coli*	O157:H7	ATCC 43895	EHEC (STEC)	+	+	+	+	+	+
3	*E. coli*	O157:H7	ATCC 8740	EHEC (STEC)	+	+	+	+	+	+
5	*E.coli*	O26:H-	NCTC 8620	EPEC	–	–	+	+	–	–
6	*E. coli*	O6 biotype 1	ATCC 25922		–	–	+	+	–	–
7	*E. coli*	O1:K1:H7	NCTC 9001	ExPEC or APEC	–	–	+	+	–	–
8	*E. coli*	O129:H11	ATCC 23542	EPEC	–	–	–	–	–	–
9	*E. coli*		NCTC 11560		–	–	–	–	–	–
4	*E. coli*		ATCC 35218		–	–	–	–	–	–
10	*E. coli*	O103	NCTC 8196	EHEC, STEC non-O157	–	–	–	–	–	–
11	*E. coli*	W3110	ATCC 27325		–	–	–	–	–	–
12	*Bacillus cereus*		ATCC 11778		–	–	+	+	–	–
13	*Klebsiella pneumonia*		ATCC 13883		–	–	+	+	–	–
14	*Pseudomonas aerouginosa*		ATCC 27853		–	–	+	+	–	–
15	*Shigella dysenteria*		NCTC 4837		–	–	–	–	–	–
16	*Listeria monocytogenes*		ATCC 19115		–	–	–	–	–	–

### Sensitivity analysis of the RPA-LFA

**Figure 3:**
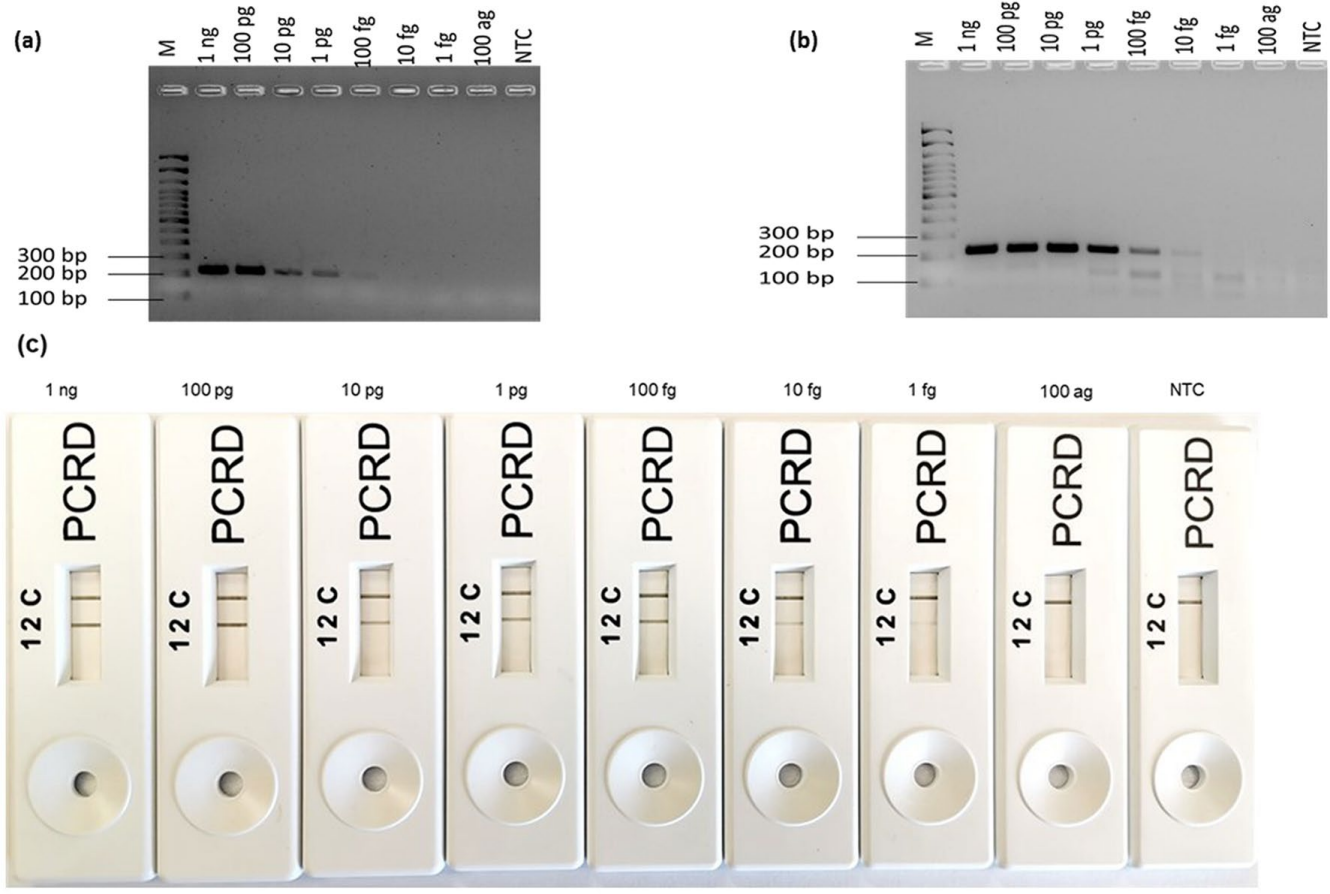
Sensitivity evaluation of RPA-LFA (by using rfbE F1/R1) to detect various DNA concentrations of *E. coli* O157:H7. From left to right 1 ng to 100 ag and non-template control (NTC). (**a**) By PCR-AGE, (**b**) by RPA-AGE assay, (**c**) by RPA-LFA.

**Figure 4:**
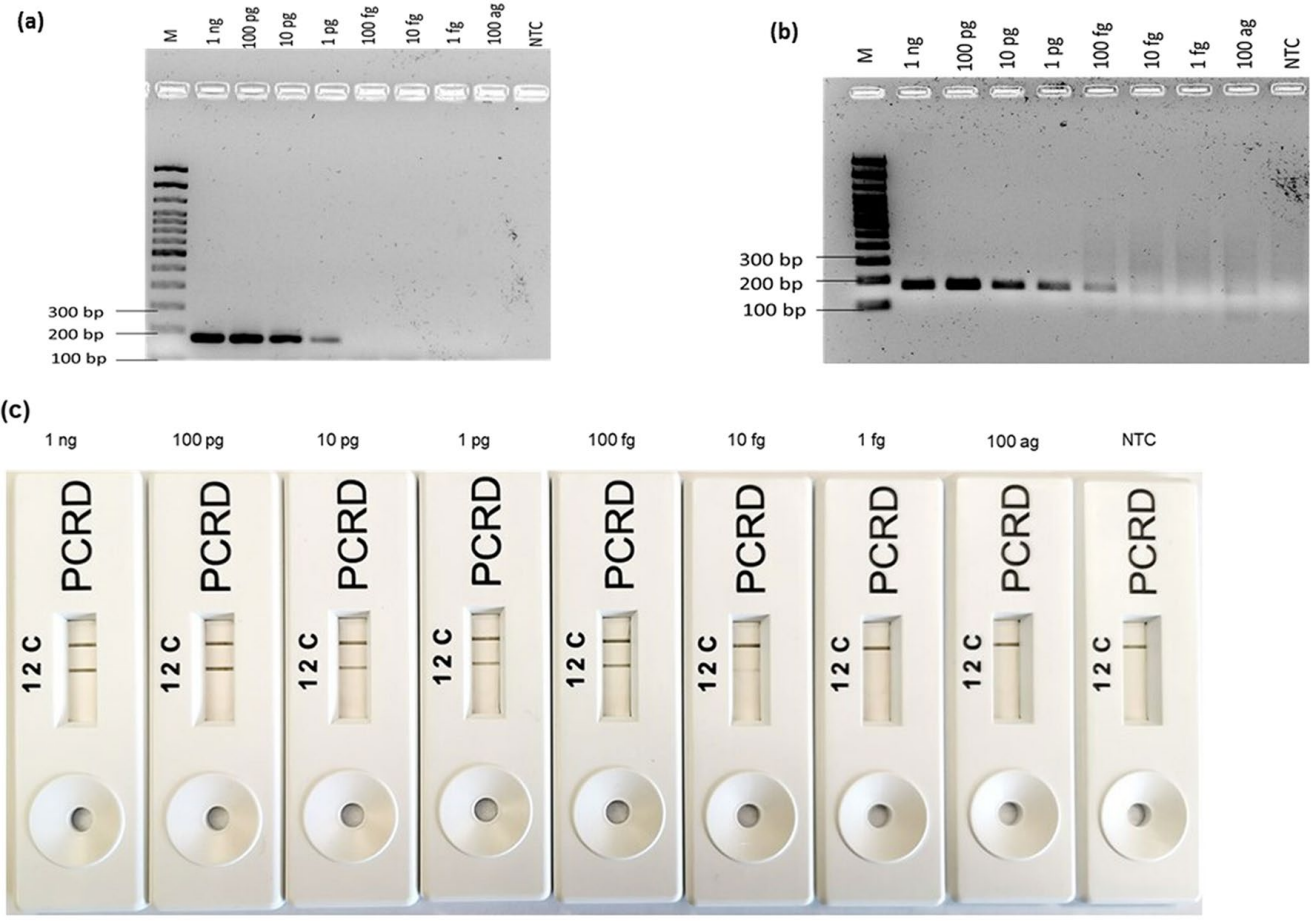
Sensitivity evaluation of the RPA-LFA using fliC F2/R2 to detect various DNA concentrations of *E. coli* O157:H7. From left to right 1 ng to 100 ag and non-template control (NTC). (**a**) By PCR-AGE, (**b**) by RPA-AGE assay, (**c**) by RPA-LFA.

**Figure 5:**
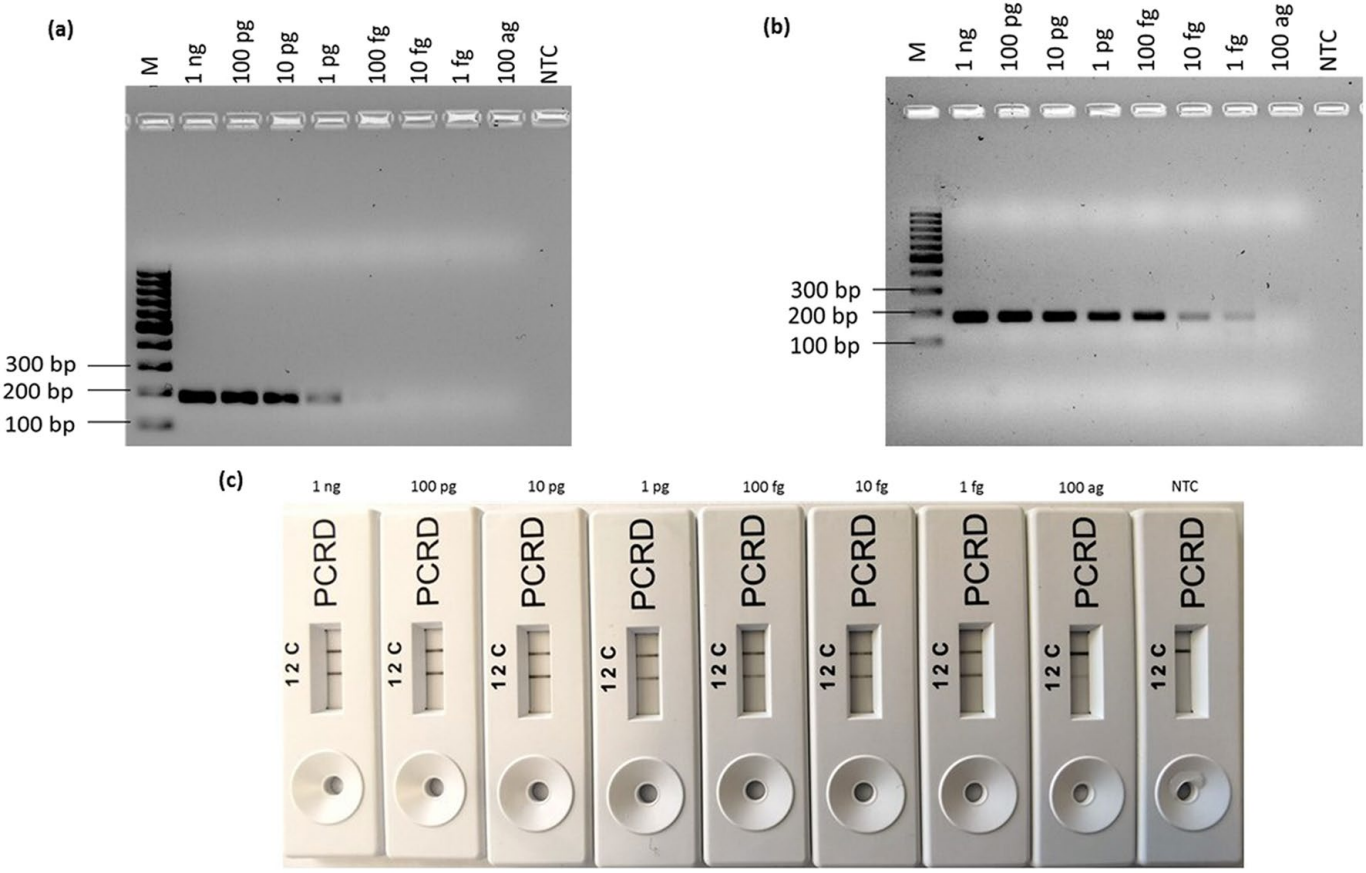
Sensitivity evaluation of the RPA-LFA using stx2 F1/R1 to detect various DNA concentrations of *E. coli* O157:H7. From left to right 1 ng to 100 ag and non-template control (NTC). (**a**) By PCR-AGE, (**b**) by RPA-AGE assay, (**c**) by RPA-LFA.

Following optimization and development of RPA-LFA targeting three different genes of *E. coli* O157:H7, evaluation of the sensitivity of LFA was performed using serial dilutions of the DNA extracted from pure *E. coli* O157:H7 cultures. Figures 3c, 4c and 5c show, from left to right, the results for the detection of different concentrations of DNA; 1 ng, 100 pg, 10 pg, 1 pg, 100 fg, 10 fg, 1 fg, 100 ag and NTC based on amplification of *rfbE*, *fliC* and *stx* genes. For the *rfbE* gene target, the RPA-LFA was able to detect as low as 1 fg of DNA (Fig. 3c); for the *fliC* gene, the detection limit was 10 fg (Fig. 4c), 10 times lower than the *rfbE* targeting RPA-LFA. DNA at a concentration as low as 100 ag was detected using RPA-LFA, targeting the *stx* gene (Fig. 5c), 10 times more sensitive than *rfbE* and 100 times more sensitive than the *fliC* gene. The colour intensity of the test line (2) was successively reduced with decreasing DNA concentration, though still readily visible. For NTC, no coloured test line (2) was observed in any RPA-LFA.

The LOD of all RPA-LFA was compared with PCR-AGE and RPA-AGE assays. The PCR method amplified the *rfbE* gene at a concentrations as low as 1 pg; a faint band was observed for 100 fg. However, the RPA-AGE assay was 10 times more sensitive than the PCR assay, with amplification of 10 fg DNA (Fig. 3a,b). The minimum concentration of the *fliC* gene amplified by PCR-AGE was 1 pg, again 10 times less sensistive than the RPA-AGE assay that successfully amplified the same gene down to 100 fg (Fig. 4a,b). For the *stx* gene, for PCR amplification, results were observed similar to those observed for the *rfbE* gene with amplification of 1 pg and a faint band for 100 fg; however, RPA-AGE was found to be 100 times more sensitive than PCR-AGE, with amplification at 1 fg (Fig. 5a,b).

### Visual assay analysis of the RPA-LFA in real samples

**Figure 6:**
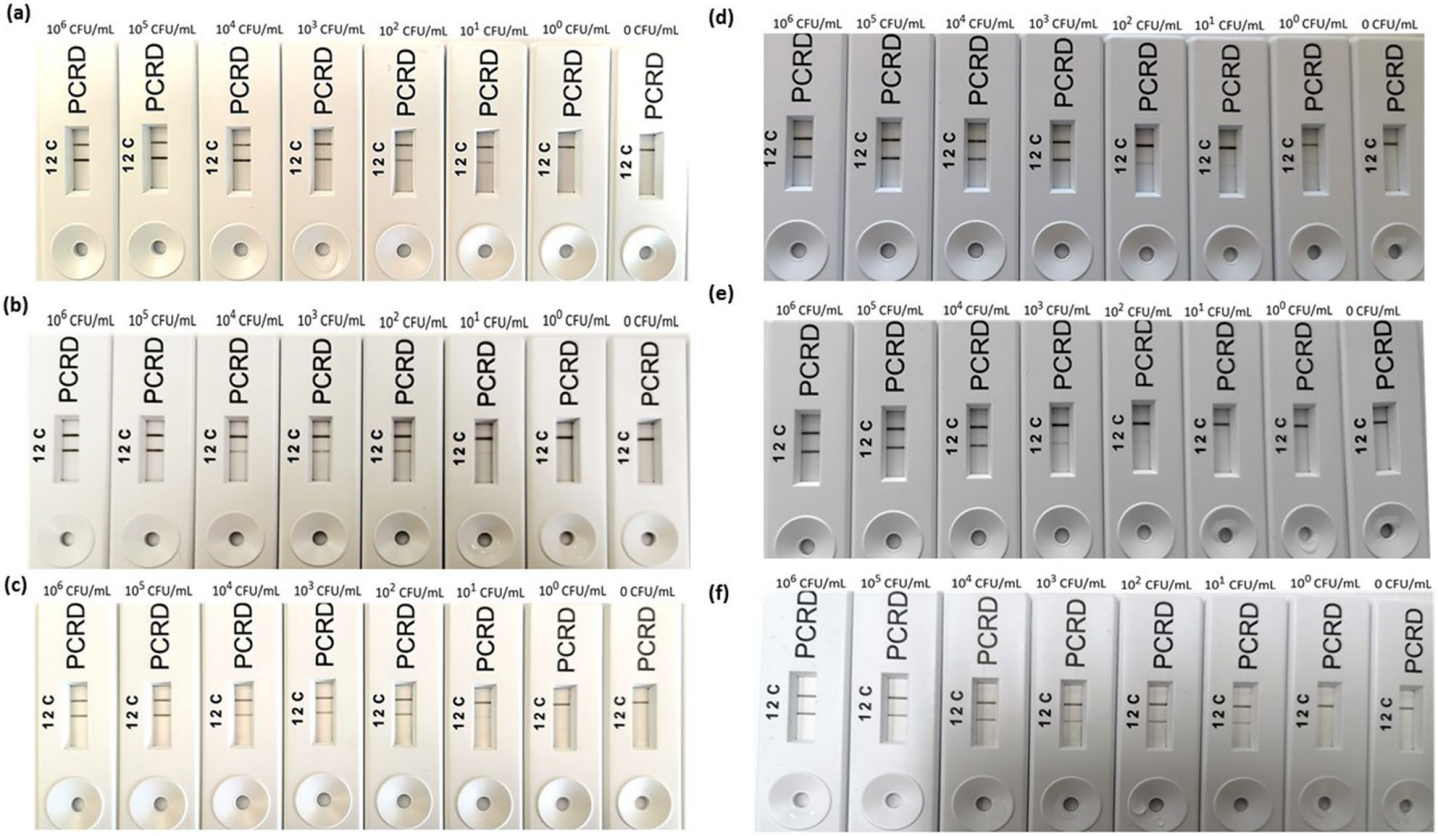
Typical images of the RPA-LFA (**a**–**c**) using rfbE F1/R1 primer set (targeting *rfbE* gene) and (**d**–**f**) stx2 F1/R1 (targeting *stx* gene) for the spiked commercial samples. From left to right, the concentrations of *E. coli* O157:H7 are 10^6^ CFU/mL, 10^5^ CFU/mL, 10^4^ CFU/mL, 10^3^ CFU/mL, 10^2^ CFU/mL, 10^1^ CFU/mL, 10^0^ CFU/mL and 0 CFU/mL. (**a**, **d**) For drinking water samples, (**b**, **e**) for milk sample, (**c**, **f**) for apple juice sample.

Following post-optimisation and validation of both RPA-LFA’s, the assay targeting the *rfbE* and *stx* genes were selected to apply for the commercial samples. Commercially available drinking water,apple juice and milk were collected and confirmed for the absence of *E. coli* O157:H7. NB containing 10^7^ CFU/mL of *E. coli* O157:H7 (1 mL) was inoculated into 9 mL of water, apple juice and milk samples followed by serial dilution in the respective samples to get final concentrations as 10^6^ CFU/mL, 10^5^ CFU/mL, 10^4^ CFU/mL, 10^3^ CFU/mL, 10^2^ CFU/mL, 10^1^ CFU/mL, 10^0^ CFU/mL. Uninoculated water, apple juice and milk were used as negative controls. *E. coli* O157:H7 was detected in all serial dilutions for all the three types of samples used with both selected RPA-LFA; no *E. coli* O157:H7 was detected in the negative controls (Fig. 6). For the *rfbE* gene target, the sensitivity of the RPA-LFA was 10^1^ CFU/mL for all the artificially spiked commercial samples (Fig. 6a–c); for the *stx* gene target, *E. coli* O157:H7 was detected at 10^2^ CFU/mL in milk, 10^0^ CFU/mL (approximately 4–5 CFU/ mL) in water and 10^1^ CFU/mL in apple juice.

## Discussion

The provision to the water and food sectors of specific and sensitive POC detection methods will significantly achieve some of the important goals from the 2030 agenda for sustainability development, adopted by all the members of the United Nation in 2015^21^. Early detection of *E. coli* O157:H7 in the surrounding environment including water and food will aid monitoring of the quality and sanitation^22^. Further, the provision of clean water for multiple purposes will significantly benefit health and well-being, especially in endemic areas or developing countries^23^. Molecular techniques have gained credence over other techniques in terms of their sensitivity and specificity^24^. However although several promising PCR methods have been developed for the detection of pathogenic *E. coli* O157:H7 in water, milk and other foods, PCR detection methods cannot be used at the POC and in resource limiting settings. In addition, PCR based techniques are expensive with slow turnaround times. Thus, a simple POC method for use at the site of sample collection, with a short detection time, high specificity and sensitivity is of significant commercial interest. The isothermal RPA technique developed by Piepenburg et al.^16^ has been the focus of interest in the last few years to overcome the limitations of PCR^25^.

In this study, RPA was coupled with lateral flow based detection bioassay (PCRD cassettes) to detect *E. coli* O157:H7 in drinking water, apple juice, and milk. The assessment of three different genes for *E. coli* O157:H7 identification using the RPA amplification coupled with LFA brings novelty to this research study. In addition, the extension of RPA-LFA assay for detection of *E. coli* O157:H7 in more commercially available products such as drinking water and apple juice has been performed for the first time.

Primer design and selection are key steps in achieving efficient and reproducible DNA amplification through RPA^18^; PrimerRPA software was chosen to design RPA primers because of its development with default filters to get specific RPA primers in silico for target sequences^20^. The *rfbE* gene encodes perosamine synthetase, involved in the expression of O-antigens in *E. coli* O157:H7^26^; the *fliC* gene encldes flagellin, involved in adherence of the pathogen to the epithelial cells. The *stx* gene encodes for Shiga-toxins which are protein synthesis inhibitors^4^. These genes were screened as they represented the most reliable and specific gene targets based on previously reported studies^27^. One primer set for each selected gene was screened to determine the effect of different primers for the RPA assay. Replicate assays were performed to avoid non-specific amplification which can be caused by primer self-complementarity. To observe the specific amplification, template and non-template controls were checked in the primary screening of all primers.

Out of the all RPA-LFA’s, targeting the *stx* and *rfbE* gene was found extremely sensitive, detecting 100 ag and 1 fg DNA of pure *E. coli* O157:H7 culture respectively. In contrast, the amplification of the *fliC* gene target could only detect as low as 10 fg DNA. This difference in sensitivity between gene targets supports the findings of the study by Liu et al.^19^ which reported a higher sensitivity of the *rfbE* compared with the *fliC* gene using a DNA microarray method. The selection of gene targets and primer set is crucial in terms of enhancing the sensitivity and specificity of the RPA method. In this study RPA-LFA assays detecting *stx* and *rfbE* were more specific (100%) than the *fliC* gene (72.8%) for *E. coli* O157:H7 detection from a mixture of foodborne and waterborne pathogens. The 100% specificity of RPA-LFA using the *rfbE* gene target confirms the results from the study conducted by Hu et al.^18^. The lower specifity using the *fliC* gene could be due to the presence of the *fliC* gene in other closely related bacterial strains^28,29^. Therefore, RPA-LFA using the *stx* and *rfbE* gene was selected as highly selective and sensitive assay for target detection.

In addition to exhibiting good specificity and selectivity, LFA does not require any sophisticated laboratory set up. The nfo enzyme works at 39 °C, producing a cut at the THF residue that generates a 3′OH group in the primary amplicons of the forward and reverse primers, and then extends the strand by displacement. The final amplified DNA molecule has two labels on opposite ends, biotin and FAM which can be detected by a sandwich format. Visualization of the product was achieved using simple lateral flow action of the amplified product towards the porous membrane from the sample well. Gold nanoparticle-conjugated anti-FAM antibody bind to the FAM molecule on the conjugation pad, which then move toward test line 2 which has an anti-biotin label to capture the biotin, producing a coloured test line, enabling visual detection. The full detection assay can be performed at 39 °C, or even held in the palm of a hand, eliminating the need for any special heating instrument to perform the assay. Furthermore, the assay can be completed within 8 min for a final visual outcome as positive or negative samples; in contrast, PCR methods take up to 2–3 h to visualize the amplified products on the gel^30^.

For the artificially spiked drinking water, apple juice and milk samples, the LOD of the RPA-LFA was down to 10^1^ CFU/mL (using the *rfb*E gene target) and 4–5 CFU/mL (using the *stx* gene target); this sensitivity is comparable to the RPA-AGE assay developed using the *stx* gene^31^. The sensitivity of the assay supports its commercial application, as the infectious dose of *E. coli* O157:H7 for human is 10 CFU/mL which is readily detectable with this RPA-LFA^32^. The originality of the study was based on the evaluation of three genes for development of RPA-LFA for detection of *E. coli* O157:H7 and its validation on more commercial food samples. The simplicity, specificity and reproducibility of this assay offers its suitability for in-field testing of water and other food samples.

## Conclusion

The *rfbE* and *stx* gene targets were found both more specific and sensitive than the *fliC* gene target in identifying the *E. coli* O157:H7 using the RPA based LFA assay. The assessment of RPA-LFA (using rfbE F1/R1 and stx2 F1/R1) for detection of target pathogen in drinking water, apple juice and milk samples was found highly selective, simple, cost-effective and sensitive, identifying 100 ag of *E. coli* O157:H7 from a mixed bacterial sample; this is 1000 times sensitive than PCR. Recombinase Polymerase Amplification can amplify DNA in 5 min at a temperature ranging from 37 to 42 °C. This technology is potentially significant for use in remote settings or resource limited locations. Enumeration of the presence of *E. coli* O157:H7 in contaminated food, drinking water, water pipelines, private water storage tanks, effluent wastewater, surface water and packed and non-packed food items can give an estimation of hygiene, which can reduce the spread of contamination. This RPA based platform will support the sustainability goals of providing clean water, safe food and healthy life to our society. Moreover, the method can be integrated with simple DNA extraction methods to make it a fully point-of-care detection tool.

## Material and methods

### Collection of bacterial isolates and their cultivation

Pure cultures of 22 bacterial strains including *E. coli* O157:H7 NCTC 12900, *E. coli* K-12 NCTC 10538, *Staphylococcus aureus* ATCC 25923, *Salmonella typhimurium* ATCC 14028,* Enterococcus faecalis* NCTC 12201 and *Shigella flexneri* ATCC 122022 were obtained from the microbial culture collection of the Royal Melbourne Institute of Technology (RMIT, Australia). *E. coli* O157:H7 strain NCTC 12900 was used as a target (positive reference) for this study and all other non-STEC O157:H7 bacterial strains (listed in Table 3) were used as negative references for the specificity test.

Bacterial strains were cultured in nutrient broth (NB) (Sigma-Aldrich, Germany) at 37 °C with shaking at 170 rpm. Growth was monitored by measuring the optical density at 600 nm (OD_600_) until it reached 0.5–0.6, at which point DNA was extracted. Enumeration of *E. coli* O157:H7 cells was performed using a plate culture method; tenfold serially diluted *E. coli* O157:H7 cultures were plated on nutrient agar (NA) (Sigma-Aldrich, Germany) and incubated at 37 °C overnight.

### Total DNA extraction

Total genomic DNA was extracted from all bacterial strains using the Quick-DNA Faecal/Soil Microbe Kit (Zymo Research, USA). Briefly, 50–100 mg wet bacterial cells pellet was resuspended in 200 µL of 1X phosphate buffer saline (PBS) followed by the manufacturer’s guidelines. The total double-stranded DNA for all bacteria was quantified by Qubit 2.0 fluorometer (Invitrogen, California, United States) and stored at − 20 °C for further use.

### Designing and online-screening of primers

The perosamine synthetase encoding *rfbE* (GenBank accession No. S83460), flagellin encoding *fliC* (GenBank accession No. U47614) and shiga toxin encoding *stx*1 and *stx*2 (GenBank accession No. NC_002695) genes were chosen as the specific target genes for *E. coli* O157:H7 detection based on previously reported studies. The primers were designed according to the TwistAmp Basic kit (TwistDX, USA) manual (30–35 bp length, 30–70% GC content, GC clamp at the 3′ end and no poly G at 5′ end is preferred). PrimedRPA, automated software was used to design RPA primers. The target gene sequences were taken from the NCBI in ‘fasta format’ and entered in the software. The selection parameters were entered and checked (100–200 bp amplicon lenght, GC content 35–55% and primer 30–35 bp). The OligoAnalyzer Tool and NCBI-BLAST was used to check the cross-binding potential of all primers to the non-target nucleotide genomes. Primers having low binding score were removed by in silico filtration and a total of eight primer sets were selected for further in vitro screening (Table 1). All the primers and probes were synthesized by Bioneer Pacific (Bioneer Corporation Daejeon, Republic of Korea) (https://pharmaboardroom.com/).

### In-vitro screening of primers by RPA-AGE assay

To screen the best primer set for *rfbE*, *fliC* and *stx* genes, 4 primer pairs for each gene, named Eco rfbE F1/R1, Eco rfbE F2/R2, Eco rfbE F3/R3, Eco rfbE F4/R4 for *rfbE,* Eco fliC F1/R1, Eco fliC F2/R2, Eco fliC F3/R3, Eco fliC F4/R4 for *fliC* and Eco stx1 F1/R1, Eco stx1 F2/R2, Eco stx2 F1/R1, Eco stx2 F2/R2 for *stx* genes (Table 1) were selected and tested using the recombinase polymerase amplification-agarose gel electrophoresis (RPA-AGE) assay. Evaluation of potential primer candidates was performed using the Twistamp Basic RPA kit (TwistDX, San Diego, USA) according to the manufacturer instructions. Briefly, the reaction was performed in 50 µL volume containing 29.5 µL of rehydration buffer (from the kit), 2.4 µL of each Eco rfbE F/R primer and Eco fliC F/R primer (480 nM), 11.2 µL NFW and 2 µL of DNA template. The mixture was added to the freeze-dried reaction pellet (in a 200 µL Eppendorf tube) and mixed by pipetting. An aliquot (2.5 µL) of 14 mM of magnesium acetate (MgOAc) was added to the lids and centrifuged briefly. The RPA tubes were incubated in a thermal cycler (Bio-Rad, California, USA) at 39 °C for 20 min. After 4 min of incubation, the reaction content was mixed by inverting the RPA tubes multiple times and the incubation continued for the remaining time. The amplified RPA products were purified using the ISOLATE II PCR and Gel Kit (Bioline, London, UK) and subsequently visualized on 2% agarose gels (Bioline, London, UK) with a 100 bp marker (Thermofisher Scientific, Waltham, USA). For each primer set, *E. coli* O157:H7 NCTC 12900 and NFW were used as positive control and NTC respectively. To overcome the contamination issue, separate locations within the same laboratory were used to set-up and purify the reactions.

### Temperature and time optimization of the RPA reaction and validation

Time and temperature optimization of the RPA assay for the three selected primer sets (Eco rfbE F1/R1, Eco fliC F2/R2 and Eco stx2 F1/R1) were carried out using the RPA-AGE assay described above. The assay was performed at different six temperatures, 37 °C, 38 °C, 39 °C, 40 °C, 41 °C and 42 °C. Similarly, the optimum time was selected by incubating the RPA reaction for 5, 10, 15, 20, 25 and 30 min. One template and non-template control was included in all sets of RPA reactions throughout. Validation of both primer sets was carried out by running a specificity test using the RPA-AGE assay. Primer cross-reactivity was tested using genomic DNA from the other bacterial pathogens, S*. aureus*,* S. flexneri*,* S. typhimurium*,* E. coli K-12* and *E. faecalis*.

### Lateral flow bioassay designing and its establishment

Postvalidation of selected three primer sets using the RPA-AGE assay, LFA was developed using rfbE F1/R, fliC F2/R2 and stx2 F1/R1 primer sets. The lateral flow assay was designed and performed according to the manufacturer’s guidelines (TwistDX, San Diego, USA). The forward primer was used without any modifications and reverse primer was labeled with biotin at the 5′ end. The nfo probe was designed manually by adding a 5′-FAM label and consists of 30 nucleotides upstream and 18 nucleotides downstream connected through a THF spacer. The C3-spacer was added at the 3′ end which is a polymerase extension blocking group (Table 2). All primer and probe sequences were purchased from Bioneer. The RPA-nfo reaction was performed in 50 µL volumea by adding 29.5 µL of rehydration buffer (from the kit), 2.1 µL of each forward and reverse primer, 0.6 µL of nfo probe, 11.2 µL of NFW, 2.5 µL of MgOAc and 2 µL of template DNA. All reagents were mixed to make a master mix except DNA template and MgOAc. The lyophilized enzyme pellet (provided in the kit) was dissolved by adding 45.5 µL of prepared master mix into the 0.2 mL RPA reaction tube. Subsequently, 2 µL of the DNA template was added to the RPA tube prior to adding 2.5 µL of magnesium acetate into the lids and tubes were closed and centrifuged briefly to initiate the RPA reaction. The RPA reaction tubes were incubated at the 39 °C for 20 min with a mixing interval of 4 min incubation (T100 Thermal cycler, Bio-Rad, California, United States).

PCRD (Abingdon Health, UK), a LF-based device was used to visualize the amplified product using the naked eye. An aliquot (6 µL) of amplified product was diluted with the 84 µL of PCRD extraction buffer (provided in the kit); an aliquot (75 µL) was loaded into the sample well. The cassette was allowed to rest for the lateral flow of liquid and the appearance of bands at the control and test lines was recorded as appropriate within 10 min (usually bands appear within 2–3 min).

### Evaluation of LF bioassay for specificity and sensitivity

The specificity evaluation of both RPA-LFA was performed using additional strains of *E. coli* O157:H7 and non-target bacterial isolates (for example S*. aureus*,* S. flexneri*,* S. typhimurium*,* E. coli K-12*,* E. faecalis*) (Table 3). The amplification sensitivity limit for Eco rfbE F1/R1, Eco fliC F2/R2 and Eco stx2 F1/R1 primers was determined using 1:10 serially diluted genomic DNA of *E. coli* O157:H7 down to 100 ag (1 ng, 100 pg, 10 pg, 1 pg, 100 fg, 10 fg, 1 fg, 100 ag). The amplification sensitivity of RPA-LFA for both primer sets (targeting different genes) was compared with RPA-AGE and PCR-AGE using a 2% agarose gel. For PCR, MyFi Mix (Bioline, London, UK) was used for reaction set up. Briefly, a total reaction volume of 50 µL was used by adding 1 µL of each primer (20 µM), 25 µL of MyFi Mix, 21 µL RNase free water and 2 µL of DNA template. For NTC, 2 µL of NFW was used as a template. The PCR cycle conditions used were 1 cycle of initial denaturation at 95 °C for 3 min, 35 cycles of denaturation at 95 °C for 15 s, annealing at 58 °C for 15 s, extension at 72 °C for 15 s and a final extension at 72 °C for 5 min.

### Assessment of RPA-LFA in spiked drinking water, apple juice and milk

Post-analysis and validation of primers (targeting different genes) with RPA-AGE, PCR-AGE and RPA-LFA, based on specificity and enhanced sensitivity confirmed that RPA-LFA based on the *rfbE* and *stx* genes was suitable for use in *E. coli* O157:H7 detection in selected commercial products, bottled drinking water, apple juice and milk. All three products were purchased from a local supermarket in Melbourne, Victoria, Australia and checked for the presence of *E. coli* O157:H7 using SMAC culturing methods. Pure bacterial culture of *E. coli* O157:H7 in nutrient broth (1 mL), containing 10^7^ CFU/mL was serially diluted in 9 mL of collected commercial samples to produce tenfold serial dilutions, 10^6^ CFU/mL, 10^5^ CFU/mL, 10^4^ CFU/mL, 10^3^ CFU/mL, 10^2^ CFU/mL, 10^1^ CFU/mL, 10^0^ CFU/mL. Cell counts per mL (CFU/mL) were assessed through plate counting by spreading 100 µL of each dilution on NA plates, followed by incubation at 37 °C overnight. Subsequently, genomic DNA was extracted from each dilution for each sample and 2 µL DNA was directly added to the RPA reaction and finally visualized using the PCRD LF device. Uninoculated commercial samples were used as negative controls for validation of the assasy for each sample.

## Supplementary Information

Supplementary Information
