# Supplementary material for: Evaluation and comparison of recombinase polymerase amplification coupled with lateral-flow bioassay for Escherichia coli O157:H7 detection using diifeerent genes
Source: Sci Rep. 2021 Jan 21;11:1881. doi: 10.1038/s41598-021-81312-6 (PMC7820579; doi:10.1038/s41598-021-81312-6)
Supplement: Supplementary file 1 — Supplementary Information [file 41598_2021_81312_MOESM1_ESM.pdf]

Supplementary Document

Evaluation and comparison of Recombinase polymerase amplification coupled with lateral-flow bioassay for *Escherichia coli* O157: H7 detection using different genes

Alka Rani[a]*, Vivek B Ravindran[a], Aravind Surapaneni[c,d], Esmaeil Shahsavari[a], Nagalakshmi Haleyur[a], Nitin Mantri[b], Andrew S Ball[a,d]

[a] *School of Science, RMIT University, Bundoora West, Victoria 3083, Australia*

[b] *The Pangenomics Group, School of Science, RMIT University, Melbourne, VIC, 3083, Australia*

[c] *South East Water, Frankston, Victoria, Australia*

[d] *ARC Training Centre for the Transformation of Australia's Biosolids Resource, RMIT University, Bundoora West, Victoria 3083, Australia*

*Correspondence: alka.rani@rmit.edu.au. Tel: +61399256594

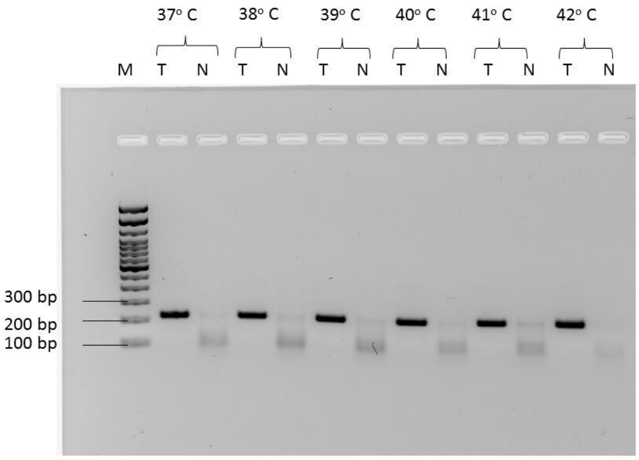

Figure S1: Temperature optimization of rfbE F1/R1 primer set by RPA-EF assay. Lane 1: Molecular marker 100 bp; from left to right, lane 2,4,6,8,10 and 12 have RPA of *E. coli* O157:H7 template (**T**) at 37° C, 38° C, 39° C, 40° C, 41° C and 42° C respectively. Lane 3, 5, 7, 9, 11 and 13 have non-template control (**NTC**) at 37° C, 38° C, 39° C, 40° C, 41° C and 42° C respectively. M= Molecular marker, T= template, N= non-template control.

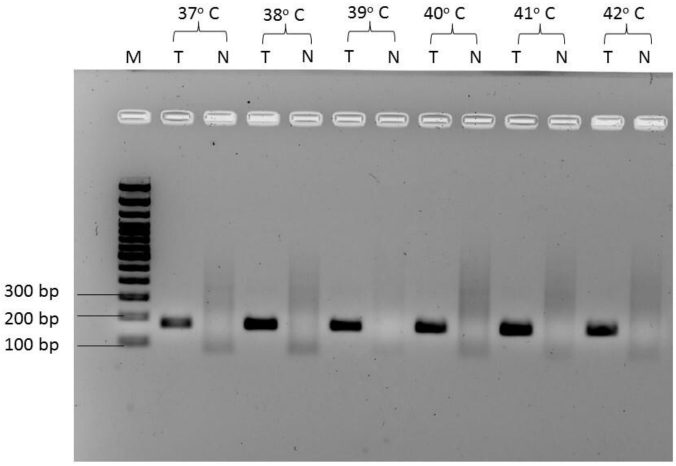

Figure S2: Temperature optimization of fliC F1/R1 primer set by RPA-EF assay. Lane 1: Molecular marker 100 bp; from left to right, lane 2, 4, 6, 8, 10 and 12 have RPA of *E. coli* O157:H7 template (**T**) at 37° C, 38° C, 39° C, 40° C, 41° C, 42° C respectively. Lane 3, 5, 7, 9, 11 and 13 have non-template control (**NTC**) at 37° C, 38° C, 39° C, 40° C, 41° C, 42° C respectively. M= Molecular marker, T= template, N= non-template control.

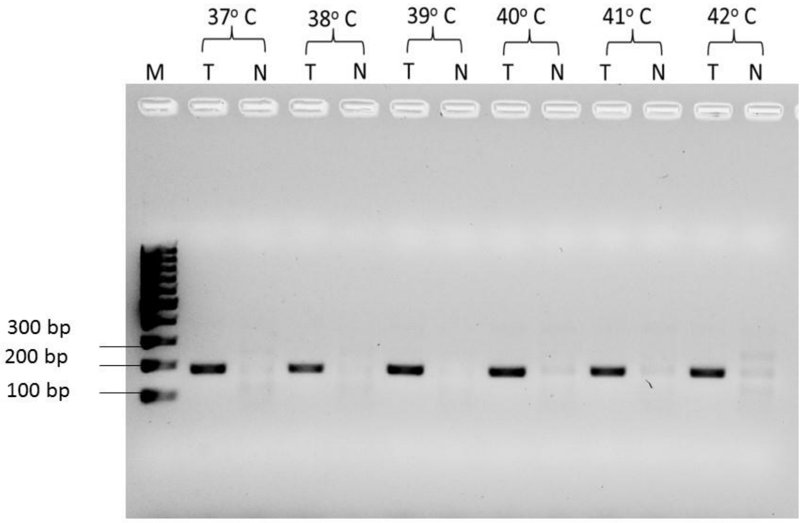

Figure S3: Temperature optimization of stx2 F1/R1 primer set by RPA-EF assay. Lane 1: Molecular marker 100 bp; from left to right, lane 2, 4, 6, 8, 10 and 12 have RPA of *E. coli* O157:H7 template (**T**) at 37° C, 38° C, 39° C, 40° C, 41° C and 42° C respectively. Lane 3, 5, 7, 9, 11 and 13 have non-template control (**NTC**) at 37° C, 38° C, 39° C, 40° C, 41° C and 42° C respectively. M= Molecular marker, T= template, N= non-template control.

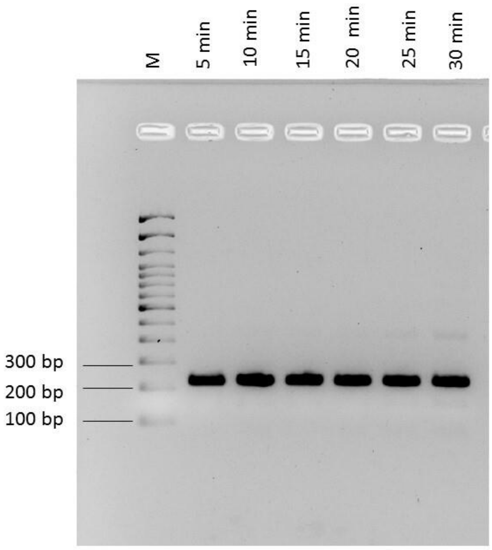

Figure S4: Optimization of reaction time for primer set rfbE F1/R1 with RPA-EF assay. Lane M: Molecular marker 100 bp, lane 2-7: different time incubation from 5 minutes to 30 minutes by using the same amount of template (2 ng).

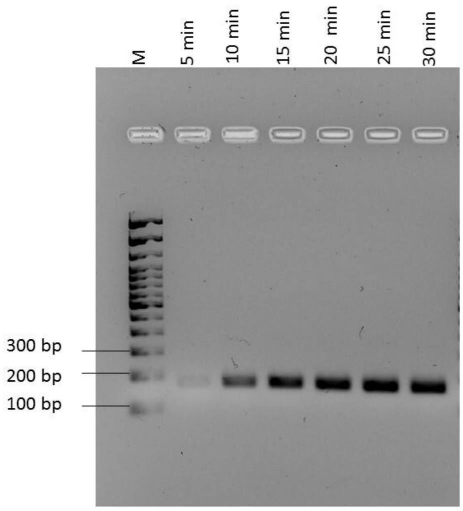

Figure S5: Optimization of reaction time for primer set fliC F2/R2 with RPA-EF assay. Lane M: Molecular marker 100 bp, lane 2-7: different time incubation from 5 minutes to 30 minutes by using the same amount of template (2 ng).

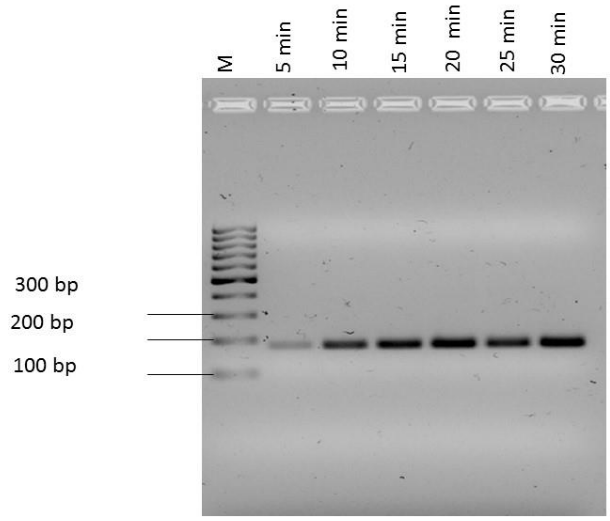

Figure S6: Optimization of reaction time for primer set stx2 F1/R1 with RPA-EF assay. Lane M: Molecular marker 100 bp, lane 2-7: different time incubation from 5 minutes to 30 minutes by using the same amount of template (2 ng).
